# Illuminating new possibilities: Effects of copper oxide nanoparticles on gastrointestinal adenocarcinoma cells in hypoxic condition

**DOI:** 10.1016/j.heliyon.2024.e31414

**Published:** 2024-05-16

**Authors:** Seyedehsaba Talebian, Bahar Shahnavaz, Mohammadhosein Shakiba, Fatemeh B. Rassouli

**Affiliations:** a Department of Biology, Faculty of Science, Ferdowsi University of Mashhad, Mashhad, Iran; b Novel Diagnostics and Therapeutics Research Group, Institute of Biotechnology, Ferdowsi University of Mashhad, Mashhad, Iran

**Keywords:** Hypoxia, Copper oxide nanoparticles, Anticancer properties, Colon adenocarcinoma, Gastric adenocarcinoma

## Abstract

Cancer remains a major global health concern, necessitating the development of novel therapeutic approaches. Hypoxia is a common characteristic of solid tumors that plays a critical role in tumor progression, making it a prime target for anticancer therapies. This study aimed to determine the effects of copper oxide nanoparticles (CuONPs) on human gastrointestinal cancer cells in hypoxic condition for the first time. Toxicity of CuONPs was evaluated on human colon and gastric adenocarcinoma cells and normal fibroblasts by alamarBlue assay. Real-time polymerase chain reaction (PCR) was performed to study the effects of CuONPs on genes involved in cell apoptosis. To elucidate the molecular mechanisms underlying the effects of CuONPs in hypoxic condition, molecular docking was conducted on HIF-1α. Results revealed dose- and cell-type-dependent toxic effects of CuONPs, as a more significant (*p* < 0.0001) decrease in viability of LoVo cells (23 %) was observed compared to MKN-45 and HDF cells. In addition, CuONPs significantly (*p* < 0.0001) reduced LoVo cell viability down to 30.2 % in hypoxic condition. Gene expression analysis revealed significant (*p* < 0.0001) overexpression of *P53* and *BAX* but downregulation of *BCL-2* and *CCND1* after treatment with CuONPs. Molecular docking indicated the preferable binding of CuONPs to the HIF-1α PAS-B domain through interaction with 15 residues with −4.8 kcal/mol binding energy. Our findings open up new possibilities for modulating HIF-1 activity and inhibiting hypoxia-induced tumor progression.

## Key summary

• CuONPs exhibited dose- and cell-type-dependent toxicity on colon and gastric adenocarcinoma cells and regulated the expression of apoptosis mediators.
• Considerable effects of CuONPs on colon adenocarcinoma cells were retained in hypoxic condition.
• Molecular docking revealed favorable interactions between CuONPs and residues within the HIF-1α PAS-B domain.

## 1 Introduction

Nanotechnology is a rapidly advancing field of science that focuses on the manipulation of nanoparticles (NPs) with unique features, such as large surface area-to-volume ratio and increased reactivity, strength, and conductivity. The applications of NPs span diverse fields including electronics, energy, biomedicine, and environmental technologies [1,2]. *N*P-based platforms have revolutionized conventional therapeutic approaches by facilitating targeted drug delivery, reducing drug concentration, and minimizing side effects. Ongoing research is focused on further optimizing the design and functionality of therapeutic NPs to enhance their specificity, stability, and efficacy [3,4]. Metal oxide NPs have attracted a lot of attention because of their wide range of medical applications in tissue engineering, immunotherapy, diagnosis, dentistry, and regenerative medicine [5]. Copper oxide nanoparticles (CuONPs) are highly attractive due to their superior optical, electrical, and magnetic properties compared to other metal oxide NPs, coupled with the abundant availability and cost-effectiveness of copper. In the realm of biomedical applications, CuONPs exhibit antibacterial, antifungal, antiviral, antidiabetic, and anticancer effects, highlighting their valuable therapeutic potential [6].

The critical importance of cancer research is underscored by high rates of cancer occurrence and mortality worldwide, affecting populations across both industrialized and developing nations [7]. Gastrointestinal cancers pose a substantial challenge to global health with high incidence and low survival rates. Colon and gastric adenocarcinomas are ranked as the third and fifth most commonly diagnosed cancers, respectively. In 2020, there were approximately 1.9 million new cases and 935,000 deaths from colon adenocarcinoma, while gastric adenocarcinoma accounted for 1.09 million new cases and 769,000 deaths worldwide [8]. Routine modalities for both colon and gastric adenocarcinoma involve surgery, chemotherapy, and radiation therapy. Nevertheless, a major barrier to the successful management of cancer is the emergence of resistant phenotypes, which can arise from genetic mutations, altered drug targets, and enhanced DNA repair mechanisms. In addition, chemical features of the tumor microenvironment including hypoxia, acidity, and oxidative stress play critical roles in multiple aspects of cancer progression particularly metastasis and therapeutic resistance [9]. Hypoxia, defined as the oxygen pressure below 10 mmHg, is a common characteristic of 90 % of solid tumors [10]. Reduced oxygen availability can be harmful to normal cells, however, cancer cells adapt to hypoxia through the activity of hypoxia-inducible transcription factor (HIF-1) [11]. HIF-1 is a basic helix-loop-helix (bHLH) transcription factor consisting of a constitutively active β subunit and an oxygen-sensitive α subunit. In well-oxygenated conditions, prolyl hydroxylation leads to von-Hippel-Lindau protein-dependent ubiquitination and rapid proteasomal degradation of HIF-1α. Hypoxia inhibits hydroxylation of HIF-1α, allowing its accumulation and translocation to the nucleus where it forms a heterodimer with HIF-1β and activates the transcription of several genes involved in angiogenesis, metastasis, immunosuppression, and the development of therapeutic resistance [12,13]. Hence, induction of HIF-1 in hypoxic condition makes it an attractive candidate for selective targeting of cancer cells, as normal cells are unlikely to be affected.

The high incidence and mortality rates of gastrointestinal malignancies necessitate the development of innovative therapeutic approaches. Given the critical role of hypoxia in tumor progression, the identification of agents that act in hypoxic condition is of significant importance. While numerous studies have reported the anticancer potential of metal oxide NPs, there is a notable gap in research regarding their effects in hypoxic condition. Therefore, this study aimed to investigate for the first time the effects of CuONPs on gastrointestinal cancer cells in hypoxic condition. To do so, the toxicity of CuONPs was evaluated on human colon and gastric adenocarcinoma cells, as well as normal fibroblasts, using alamarBlue assay in normoxic and hypoxic conditions. The effects of CuONPs on genes involved in cell apoptosis were then investigated using real-time polymerase chain reaction (PCR). Furthermore, to elucidate the molecular mechanism underlying the effects of CuONPs in hypoxic condition, molecular docking was conducted to predict the interactions between CuONPs and HIF-1α.

## 2 Materials and methods

### 2.1 Cell treatment and viability assay

Human colon and gastric adenocarcinoma cells (LoVo and MKN-45 cell lines, respectively) and human dermal fibroblasts (HDF cell line) were obtained from the Pasteur Institute in Tehran, Iran. LoVo cells express carcinoembryonic antigen and MYB, c-MYC, H-RAS, and FOS oncoproteins and are capable of forming tumors when transplanted into nude mice [14]. MKN-45 cells are capable of expressing wild-type genes *P53*, *P16*, and *P15,* with mutations reported in the oncogenes c-MET and E-cadherin [15]. Dulbecco's modified Eagle's medium (Gibco) was used for the culture of MKN-45 and HDF cells, whereas Roswell Park Memorial Institute-1640 (Capricorn) was employed for the growth of LoVo cells. The culture media for all cell types consisted of 10 % fetal bovine serum (Gibco) and 1 % penicillin-streptomycin. For normoxic incubation, cells were maintained at 37 °C with 5 % CO_2_ and 21 % O_2_ in air (Memmert incubator). For hypoxic condition, LoVo cells were placed in a triple incubator with a gas mixture consisting of 93 % N_2_, 5 % CO_2_, and 2 % O_2_ (Binder incubator).

The cytotoxicity of CuONPs was evaluated by determining the half-maximal inhibitory concentration (IC_50_) using an alamarBlue assay [16]. LoVo and MKN-45 cells were seeded at 14,000 cells per well in 96-well plates, while HDF cells were seeded at 10,000 cells per well. Followed by over-night incubation, cells were exposed to CuONPs at concentrations of 50, 100, and 200 μg/ml, all prepared using a complete medium immediately before use. To note, untreated cells served as control, and viability assay was performed for each cell type at least three times. After 24 h of treatment, alamarBlue solution (0.1 mg/ml, Sigma-Aldrich) was added to each well (10 % v/v) and cells were incubated at 37 °C in the dark for 3 h. The absorbance (A) was then measured at 600 nm (BioTek spectrophotometer) and the percentage of cell viability was calculated using the following equation: (100-(AT-AU)/(AB-AU)) × 100, where AT, AU, and AB were the absorbances of treated cells, untreated cells, and blank control, respectively.

### 2.2 Gene expression analysis

To determine the effects of CuONPs on the expression of apoptosis mediators, real-time PCR was used. Briefly, the total RNA was extracted from cells upon 24 h treatment with 100 μg/ml CuONPs using isopropanol and chloroform (Merck) according to the manufacturer's protocol (DENAzist Asia). The purity of extracted RNA was assessed by spectrophotometry at 260 nm and 280 nm (Nanodrop 2000 Thermo instrument). Complementary DNAs (cDNAs) were synthesized by M-MuLV reverse transcriptase following the manufacturer's instruction (Parstous). The fidelity of reverse transcription was confirmed by PCR using TBP primers, followed by 1 % agarose gel electrophoresis. Real-time PCR was performed in iQ5 real-time PCR detection system (Bio-Rad) using SYBR green master mix (BioFact) and primers listed in Table 1. To note, TBP transcripts were used as internal control, and normalized values were plotted as relative fold change over untreated cells. The following PCR protocol was performed: denaturation at 95 °C for 5 min following 35 amplification cycles of denaturation at 95 °C for 20 s, annealing at 58 °C for 30 s, and extension at 72 °C for 30 s, and after additional extension step at 72 °C for 5 min, melting step was passed by gentle heating from 72 °C to 95 °C for 15 s.

**Table 1:** Primer sequences and product lengths employed in the current study.

Gene name	Forward (5′ to 3′)	Reverse (5′ to 3′)	Product size (bp)
*TBP*	ACAACAGCCTGCCACCTTA	GAATAGGCTGTGGGGTCAGT	120
*P53*	GTTCCGAGAGCTGAATGAGG	TTATGGCGGGAGGTAGACTG	123
*BAX*	GGACGAACTGGACAGTAACATGG	GCAAAGTAGAAAAGGGCGACAAC	150
*BCL-2*	GATGACTGAGTACCTGAACCG	CAGAGACAGCCAGGAGAAATC	124
*CCND1*	TGAAGGAGACCATCCCCCTG	TGTTCAATGAAATCGTGCGG	151

### 2.3 Molecular docking

The potential binding interactions between CuONPs and HIF-1α were defined by molecular docking. The crystal structure of HIF-1α (PDB ID: 4H6J) was obtained from PDB Bank (http://www.rcsb.org/pdb) and extracted using PyMOL. The structure of monoclinic CuONPs was constructed as recently reported [17], and energy minimization with the universal force field was performed by Avogadro software. CB-Dock (https://cadd.labshare.cn/cb-dock2/index.php), a web server based on AutoDock Vina 1.2.0, was used to optimize molecular structures and perform docking. Results were evaluated by the free energy of binding, and the outcomes were visualized in 2D and 3D diagrams using CB-Dock and LigPlot programs.

### 2.4 Statistical analysis

The data were assessed for normality using the Shapiro-Wilk test. Statistical differences were determined using Dunnett's multiple comparison and one-way ANOVA in GraphPad Prism (version 8.4.3). The results are presented as mean ± SD. Statistical significance was considered for p values < 0.05, <0.01, <0.001, and <0.0001.

## 3 Results

**Fig. 1:**
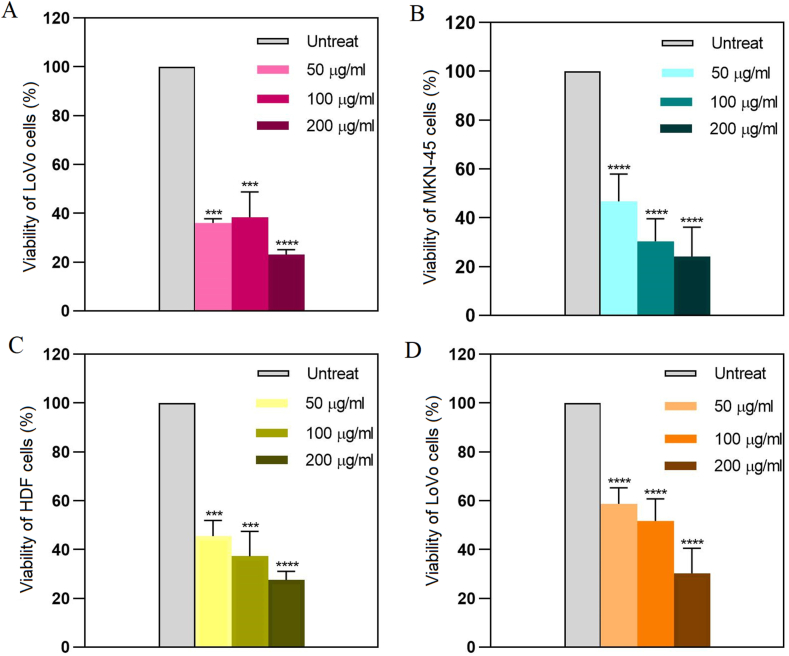
Dose-response curves demonstrating the effects of CuONPs on cell viability. alamarBlue assay was performed upon 24 h treatment of (A) LoVo cells, (B) MKN-45 cells, and (C) HDF cells with CuONPs. Viability of LoVo cells was also assed upon 24 h treatment in (D) hypoxic condition. Results are presented as the mean ± standard deviation (SD). Statistical significance is denoted by asterisks: *p < 0.05, **p < 0.01, ***p < 0.001, and ****p < 0.0001, indicating a significant difference compared to untreated cells.

**Fig. 2:**
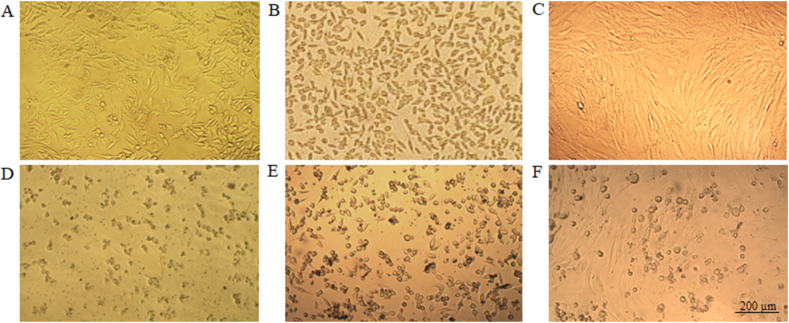
Morphological alterations of cells upon treatment with CuONPs. Phase contrast photomicrographs of (A) untreated LoVo cell, (B) untreated MKN-45 cells and (C) untreated HDF cells, and also (D) treated LoVo cells, (E) treated MKN-45 cells and (F) treated HDF cells with CuONPs for 24 h.

To evaluate anticancer potential of CuONPs, LoVo, MKN-45 and HDF cells were treated with CuONPs (NanoSadra) at concentrations of 50, 100, and 200 μg/ml for 24 h. Fig. 1 presents the results of the viability assay in three cell lines. As indicated, CuONPs induced dose- and cell-type-dependent toxic effects. It is noteworthy that upon treatment of LoVo cells (Fig. 1-A) at concentrations of 50 (*p* = 0.0002), 100 (*p* = 0.0003) and 200 (*p* < 0.0001) μg/ml, more significant decrease in viability (23 %) was observed compared to MKN-45 cells (Fig. 1-B) and non-cancerous HDF cells (Fig. 1-C). Accordingly, LoVo cells were selected to determine whether CuONPs have the potential to induce toxic effects in hypoxic condition as well. After 24 h treatment with CuONPs and incubation in hypoxic condition, viability of LoVo cells was determined. As shown in Fig. 1-D, CuONPs significantly (*p* < 0.0001) reduced cell viability down to 30.2 % in hypoxic condition. Morphological observations confirmed the findings of viability assay, revealing a diminished number of adherent and viable LoVo (Fig. 2-A and -D), MKN-45 (Fig. 2-B and -E) and HDF (Fig. 2-C and –F) cells following treatment with CuONPs compared to untreated cells. To note, the viability of LoVo and HDF cells was also evaluated upon treatment with sutent (NanoAlavnd), an anticancer drug which was considered as positive control in our study. The IC_50_ values of CuONPs in LoVo, MKN-45 and HDF cells were calculated as 17.05, 39.79 and 36.22 μg/ml, respectively. The IC_50_ values of sutent were determined as 42.59 and 25.74 μg/ml after 24 h treatment of LoVo and HDF cells, respectively. In addition, the IC_50_ of CuONPs and sutent in LoVo cells in hypoxic condition were as 81.7 and 14.06 μg/ml, respectively.

**Fig. 3:**
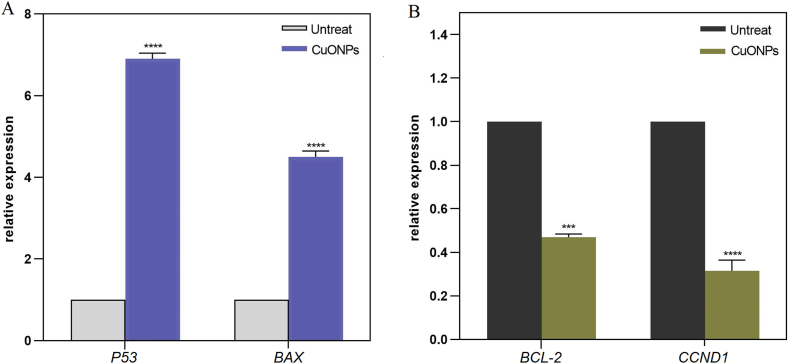
Quantification of gene expression by real-time PCR. Relative fold changes of (A) *P53* and *BAX*, as well as (B) *BCL*-*2* and *CCND1*, were calculated and normalized for untreated cells. The results are presented as the mean ± standard deviation (SD). Statistical significance is denoted by asterisks: *p < 0.05, **p < 0.01, ***p < 0.001, and ****p < 0.0001, indicating a significant difference compared to untreated cells.

To better investigate anticancer effects of CuONPs, the expression of apoptosis mediators was detected by real-time PCR. As shown in Fig. 3-A, treatment of LoVo cells with 100 μg/ml CuONPs for 24 h resulted in significant (*p* < 0.0001) over expression of *P53* and *BAX*. In addition, CuONPs significantly (*p* < 0.001) downregulated *BCL*-*2* and *CCND1* compared to untreated control cells (Fig. 3-B).

**Fig. 4:**
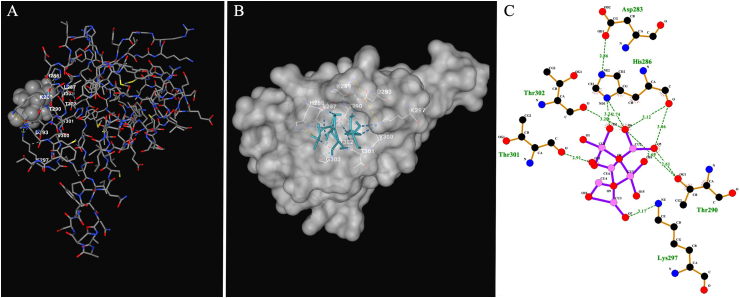
Molecular docking diagrams of monoclinic CuONPs binding to HIF-1α. CuONPs show −4.8 kcal/mol binding energy in docking with the HIF-1α PAS-B domain. The interaction involved 15 residues and the cavity volume was 13 Å. (A) 3D image of the interaction in which HIF-1α and CuONPs are shown in Licorice and Spacefill styles, respectively. (B) 3D image of the interaction in which HIF-1α and CuONPs are shown in Surface and Licorice styles, respectively. (C) 2D image of the interaction in which hydrogen bonds are visualized. Images were generated with CB-Dock and LigPlot programs.

Elucidating the molecular mechanism underlying the effects of CuONPs in hypoxic condition, molecular docking was performed to analyze the interactions between CuONPs and HIF-1α. The preferable binding of CuONPs to HIF-1α PAS-B domain was obtained through ASP285, HIS286, LEU287, LYS289, THR290, HIS291, ASP293, MET294, LYS297, GLN299, VAL300, THR301, THR302, GLY303 and GLN304 bond formation (Fig. 4-A). Hydrogen bonds were formed with HIS286, THR290, LYS297, THR301, THER302 and ASP283. To note, CuONPs show −4.8 kcal/mol binding energy in docking with HIF-1α PAS-B domain, the cavity volume was 13 Å, the center was −28, −18, −31 and docking was as 19.19.19 Å (Fig. 4-B and –C).

## 4 Discussion

Therapeutic platforms based on NPs have emerged as revolutionary approaches in cancer treatment, offering satisfying clinical outcomes while minimizing side effects [18,19] Gastrointestinal cancers are among the leading causes of cancer-related mortality worldwide. Since early diagnosis of these malignancies is often challenging due to the absence of clear symptoms, most patients have a poor prognosis of less than a year [8].

Hypoxia is a prevalent characteristic of solid tumors that plays crucial roles in tumor progression by inducing metastasis, immunosuppression and therapy resistance [12]. Accordingly, targeting HIF-1α as the key mediator of hypoxia adaptation presents promising opportunities for the development of more effective anticancer treatments. In the present study, we investigate for the first time whether CuONPs have the potential to induce anticancer effects on human gastrointestinal cancer cells in hypoxic condition.

The cytotoxicity of CuONPs was assessed on three cell lines, and findings revealed dose- and cell type-dependent effects, as higher toxicity was detected in LoVo cells when compared to MKN-45 cells and normal fibroblasts. In consistent with our results, it has been reported that CuONPs induced apoptosis in ovarian cancer cells while causing negligible toxicity on human normal skin fibroblasts [20]. Similarly, biosynthesized CuONPs had good cytotoxicity against human breast and lung carcinoma cells with a minimum toxic effect on normal mouse fibroblasts [21]. In another attempt, promising anticancer activity of CuONPs was observed by affecting human cervical cancer cells without toxicity on human embryonic kidney cells [22]. Discriminative cytotoxic effects of biogenic CuONPs have been reported on human breast carcinoma cells compared with human foreskin fibroblasts [23]. Moreover, comparing cytotoxicity of bimetallic ZnO/CuONPs on two cancer cell lines revealed significant toxicity on melanoma cells, while effects on lung adenocarcinoma cells was relatively low [24].

Effects of CuONPs were also investigated on the expression of apoptosis mediators, and results revealed considerable upregulation of *P53* and *BAX*, while *BCL*-*2* and *CCND1* were negatively regulated. P53 tumor suppressor is a critical regulator of cell cycle progression that also induces apoptosis via regulating the expression of pro-apoptotic BAX and anti-apoptotic BCL-2 proteins. Activation of BAX leads to mitochondrial permeabilization and activates caspases, which ultimately leads to apoptosis. Conversely, overexpression of BCL-2 inhibits apoptosis and promote the survival of cancer cells [25]. CCND1, another key regulator of the cell cycle, plays a central role in cancer pathogenesis by driving uncontrolled cellular proliferation [26]. Consistent with our results, CuONPs have demonstrated the ability to rebalance pro- and anti-apoptotic proteins and induce cell cycle arrest. For instance, it has been reported that CuONPs triggered apoptosis in human lung adenocarcinoma cells by increasing the activity of *P53* [27]. Moreover, CuONPs inhibited cell growth and induced apoptosis in various cancer cells by regulating the expression of *BCL-2*, *BAX,* and *CCND1* [13,27,28].

Evaluating the efficacy of anticancer agents in hypoxic condition enables accurate assessment of their therapeutic potential against advanced cancers exhibiting hypoxia-mediated treatment resistance [29]. Results of the present study indicated for the first time, considerable toxic effects of CuONPs on colon adenocarcinoma cells in hypoxic condition. To elucidate the molecular mechanism underlying this observation, molecular docking was conducted and the interactions between CuONPs and HIF-1α were studied. HIF-1α and HIF-1β subunits are classified as members of the bHLH/Per-Arnt-Sim (bHLH/PAS) family of transcription factors and have two PAS domains (PAS-A and PAS-B). PAS domains mediate heterodimerization between HIF-1α and HIF-1β, while the basic region is crucial for binding to the HREs-DNA motif of target gene promoters. Given the importance of the transcriptional activity of HIF-1, lots of efforts have been dedicated to intervening HIF-1 regulating events including its nuclear translocation, heterodimerization, and DNA binding [30]. To date, acriflavine is the only small molecule that has been introduced as a selective inhibitor of the HIF-1α PAS-B domain, which allosterically blocks its dimerization with HIF-1β [31]. Our findings that revealed favorable interactions between CuONPs and residues within the HIF-1α PAS-B domain open up new possibilities for modulating HIF-1 activity and inhibiting hypoxia-induced tumor progression.

## 5 Conclusion

Findings of the present study demonstrated dose- and cell type-dependent toxicity of CuONPs, along with the regulation of apoptosis mediators. Interestingly, we indicated for the first time, the considerable effects of CuONPs on colon adenocarcinoma cells in hypoxic condition, and molecular docking revealed favorable interactions between CuONPs and residues within the HIF-1α PAS-B domain, which explain to some extent, observed effects in hypoxic condition. Current findings open up new possibilities for inhibiting cancer cell viability and tumor progression in hypoxic condition. Complementary investigation on animal models is warranted to fully evaluate the *in vivo* anticancer efficacy of CuONPs against hypoxic cancers.

## Funding information

This study was financially supported by 10.13039/501100003121Ferdowsi University of Mashhad, Mashhad, Iran (Grant number: 55887).

## Ethics approval and consent to participate

Not applicable.

## Human and animal rights

No animals/humans were used for studies that are the basis of this research.

## Data availability statement

The data that support the findings of this study are available on request from the corresponding authors.

## CRediT authorship contribution statement

**Seyedehsaba Talebian:** Software, Investigation, Formal analysis. **Bahar Shahnavaz:** Supervision, Project administration. **Mohammadhosein Shakiba:** Writing – original draft, Formal analysis. **Fatemeh B. Rassouli:** Writing – review & editing, Supervision, Project administration, Conceptualization.

## Declaration of competing interest

The authors declare that they have no known competing financial interests or personal relationships that could have appeared to influence the work reported in this paper.
