# Supplementary material for: Illuminating new possibilities: Effects of copper oxide nanoparticles on gastrointestinal adenocarcinoma cells in hypoxic condition
Source: Heliyon. 2024 May 16;10(10):e31414. doi: 10.1016/j.heliyon.2024.e31414 (PMC11133906; doi:10.1016/j.heliyon.2024.e31414)
Supplement: Multimedia component 1 [file mmc1.pdf]

Form to confirm authorship changes for *Heliyon*

This form must be ***signed by all authors*** when there is a change in authorship which includes changes to any of the following items: author name(s), order of the authors, the corresponding author(s), the addition of authors, the removal of authors and changes in affiliation.

By personally signing this note, **all** authors confirm that: I) the changes are in accordance with their scientific contribution, II) they agree with all the changes and III) confirm that the authorship list conforms to the authorship criteria outlined on *Heliyon*'s ethics page. IV) it is the responsibility of the corresponding author to get the signature from all co-authors accepting the change. In case of any ethic violation/malpractice in the signature, the corresponding author is accountable. The completed form should be returned along with the final/revised manuscript to proceed further with the manuscript. Manuscripts for which incomplete forms have been submitted will be rejected within 5 working days.

Please include the author's name in the correct order, followed by author's who should be removed from the manuscript.

Any disputes on the authorship list and contributions need to be resolved by the involved scientists and *Heliyon* will only proceed with the evaluation of the manuscript once we receive confirmation, through this form, that such an agreement between the authors has been reached.

***Heliyon* will not accept changes to the authorship list in the late stages of the editorial process (when a paper is in Accept in Principle stage, acceptance or after publication)**

Manuscript number: HELIYON-D-23-57453

Article title: Illuminating new possibilities: Effects of copper oxide nanoparticles on gastrointestinal adenocarcinoma cells in hypoxic condition

Complete new author list: Seyedehsaba Talebian, Bahar Shahnavaz, Mohammadhosein Shakiba, Fatemeh B. Rassouli

Date: 25/04/2024

#	First name	Last name	Dept. & Institution name	Institutional email address	Order change (Y/N)	Addition / Deletion	Change in Author name (Y/N)	Affiliation Change (Y/N)	Reason for the change	Signature
1	Seyedehsaba	Talebian	Department of Biology, Faculty of Science, Ferdowsi University of Mashhad,	talebiansaba@mail.um.ac.ir	NO	NO	NO	NO		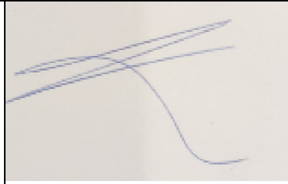

			Mashhad, Iran							
2	Bahar	Shahnavaz	Department of Biology, Faculty of Science, Ferdowsi University of Mashhad, Mashhad, Iran	shahnavaz@um.ac.ir	NO	NO	NO	NO		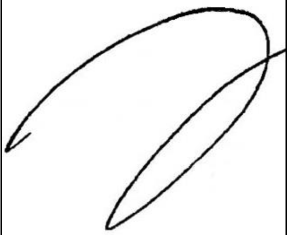
3	Mohammad hosein	Shakiba	Department of Biology, Faculty of Science, Ferdowsi University of Mashhad, Mashhad, Iran	shakiba@mail.um.ac.ir	NO	NO	NO	NO		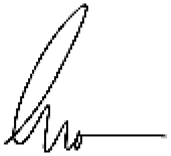
4	Omid	Rahmani	NanoSadra Company, Mashhad, Iran	info@nanosadra.com	NO	YES	NO	NO	Removal due to personal request	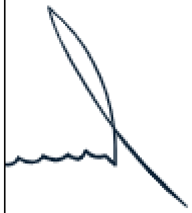
5	Fatemeh	B. Rassouli	Novel Diagnostics and Therapeutics Research Group, Institute of Biotechnology, Ferdowsi University of Mashhad, Mashhad, Iran	behnam3260@um.ac.ir	NO	NO	NO	NO		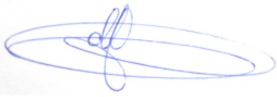
